# Histological findings in infants with Gastrointestinal food allergy are associated with specific gastrointestinal symptoms; retrospective review from a tertiary centre

**DOI:** 10.1186/s12907-015-0012-6

**Published:** 2015-06-16

**Authors:** Neil Shah, Ru-Xin Melanie Foong, Osvaldo Borrelli, Eleni Volonaki, Robert Dziubak, Rosan Meyer, Mamoun Elawad, Neil J. Sebire

**Affiliations:** Paediatric Gastroenterology Department, Great Ormond Street Hospital, London, WC1N 3JH United Kingdom; Histopathology Department, Great Ormond Street Hospital, London, United Kingdom; Institute of Child Health/UCL, London, WC1N 1EH UK

**Keywords:** Endoscopy, Infant, Food allergy, Biopsy, Histopathology, Eosinophil

## Abstract

**Background:**

Gastrointestinal food allergy (GIFA) occurs in 2 to 4 % of children, the majority of whom are infants (<1 year of age). Although endoscopy is considered the gold standard for diagnosing GIFA, it is invasive and requires general anaesthesia. Therefore, we aimed to investigate whether in infants with GIFA, gastrointestinal symptoms predict histological findings in order to help optimise the care pathway for such patients.

**Methods:**

All infants <1 year of age over a 20 year period who underwent an endoscopic procedure gastroscopy or colonoscopy for GIFA were evaluated for the study. Symptoms at presentation were reviewed and compared with mucosal biopsy histological findings, which were initially broadly classified for study purposes as “Normal” or “Abnormal” (defined as the presence of any mucosal inflammation by the reporting pathologist at the time of biopsy).

**Results:**

Of a total of 1319 cases, 544 fitted the inclusion criteria. 62 % of mucosal biopsy series in this group were reported as abnormal. Infants presenting with diarrhoea, rectal (PR) bleeding, irritability and urticaria in any combination had a probability >85 % (OR > 5.67) of having abnormal histological findings compared to those without. Those with isolated PR bleeding or diarrhoea were associated with 74 % and 68 % probability (OR: 2.85 and 2.13) of an abnormal biopsy, respectively. Conversely, children presenting with faltering growth or reflux/vomiting showed any abnormal mucosal histology in only 50.8 % and 45.3 % (OR: 1.04 and 0.82) respectively.

**Conclusions:**

Food allergy may occur in very young children and is difficult to diagnose. Since endoscopy in infants has significant risks, stratification of decision-making may be aided by symptoms. At least one mucosal biopsy demonstrated an abnormal finding in around half of cases in this selected population. Infants presenting with diarrhoea, PR bleeding, urticaria and irritability are most likely to demonstrate abnormal histological findings.

## Background

Gastrointestinal food allergy (GIFA) is increasing in prevalence and usually affects very young children [1]. Approximately 2-4 % of children between the ages of 0-3 years are diagnosed with food allergy [2, 3] and up to 60 % of these children display gastrointestinal symptoms such as abdominal pain, poor appetite, vomiting and diarrhoea. Other children may present with symptoms affecting skin, such as eczema, catarrhal problems or even anaphylaxis. Clinically, symptoms are often very pronounced and warrant investigations to eliminate other diagnoses before food allergy is considered [2, 4, 5]. Normally, the mucosal barrier in the gastrointestinal (GI) tract develops an “oral tolerance” to food antigens ingested [6]. However, in children with food allergy, this mechanism is believed to fail, resulting in allergic sensitisation and elicitation of allergy-type responses [6, 7]. This reaction, which can manifest as a wide range of different symptoms, can be classified as immunoglobulin E (IgE)-mediated allergy, non-IgE mediated allergy or mixed IgE and non-IgE allergy [8]. Gastrointestinal food allergies (GIFA) are generally considered as non-IgE mediated, but eosinophilic dominant gastrointestinal disorders may be mixed IgE and non-IgE allergies. The most common age of presentation of non-IgE mediated allergies affecting the gut is in children under the age of one year, with cow’s milk, soy protein, hens’ egg and wheat being the most frequent causative foods [2, 4, 5, 9, 10].

**Fig. 1:**
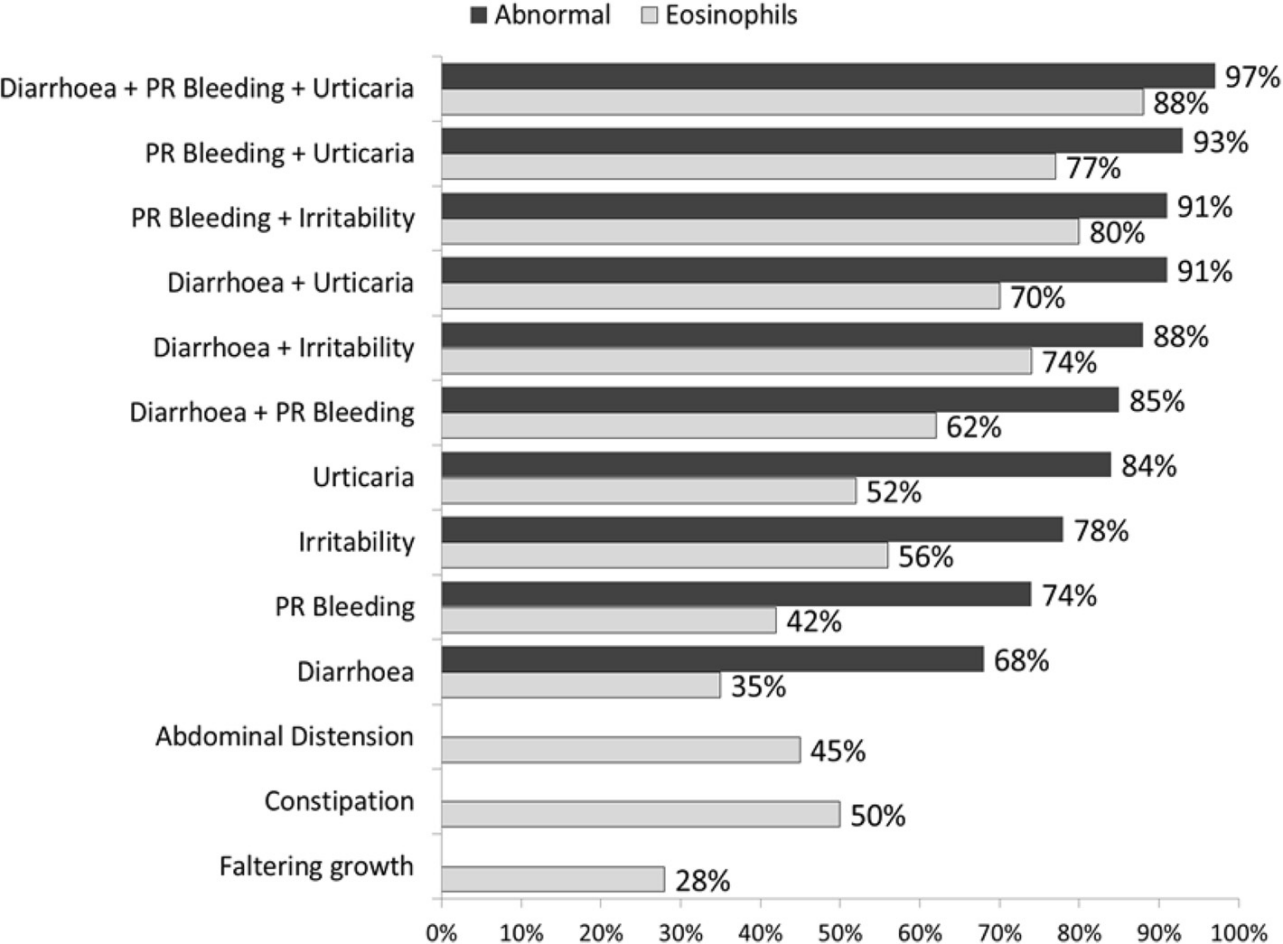
Frequencies of abnormal biopsy findings for single symptoms and combinations of symptoms common to two regression models

The immunopathology of non-IgE mediated GIFA is still not fully understood, which makes diagnosis and management difficult, often requiring an elimination diet followed by food challenge [8, 11]. Endoscopy and biopsy has become increasingly important, with some considering endoscopic biopsy as the gold standard since it is relatively objective and may provide information regarding possible mechanisms. [4, 7] For example, in eosinophilic oesophagitis, the histological appearance defines the diagnosis [12]. However, endoscopy for very young children is often limited to specialised centres and involves general anaesthesia, requiring administration by paediatric anaesthetists, and procedural risks such as intestinal perforation [13, 14]. There are no studies investigating gastrointestinal symptoms in relation to histological features in infant GIFA [15]. Hence, the aim of this study was to investigate whether specific symptoms are associated with abnormal histological findings in endoscopic biopsies obtained from children with GIFA in order to optimise care pathway decision making.

## Methods

Routinely collected data was reviewed from children under the age of one-year referred to a tertiary paediatric gastroenterology centre during the study period (June 1987 to August 2007), who had undergone endoscopic biopsy. Jejunal biopsies performed by the now historical procedure of Crosby capsule (common in the early years of our study) were excluded, and we also excluded children biopsied for other indications unrelated to GIFA. For all cases clinical symptoms were assessed in relation to histopathological findings based on contemporaneous biopsy reports. A single researcher extracted data according to predefined objective criteria.

All biopsies were reported by specialist paediatric histopathologists from the same tertiary centre. For the purposes of this study, histopathological findings were coded as either “Normal” or “Abnormal” (presence of any significant abnormal finding at any biopsy site including acute or chronic inflammation, with or without increased mucosal eosinophil density [16], or other pathologies such as partial villous atrophy or *Helicobacter pylori* see Fig. 1). Chronic inflammation with predominantly excess mucosal eosinophil density was considered most suggestive of food allergy in this cohort of young children [12, 17, 18].

Data were analysed using IBM SPSS Statistics for Windows, Version 22 (Armonk, NY). Continuous variables were presented as medians with interquartile ranges and categorical variables as frequencies and percentages. Mann-Whitney *U* test and chi-square test were used to examine the differences between groups.

For all symptoms in isolation (in cases when the patient presented with only one symptom) Positive Predictive Values (PPV) of abnormal biopsy findings were calculated. Multiple logistic regression was used to investigate the relationship between biopsy findings and symptoms with adjustment for potential confounders of age and gender. Based on logistic regression models, probabilities of abnormal biopsy findings were calculated for combinations of symptoms using the median age. Goodness of fit of logistic regression models was based on Hosmer-Lemeshow test. All tests were two-tailed and significance level was set to 0.05. The study was approved by the local Research Ethics Committee (Bloomsbury REC). All data was retrospective and identified by study number only and individual patient consent was not required for data inclusion. The study conformed to the Helsinki declaration regarding research performance.

## Results

Of 1319 infants undergoing endoscopic biopsies, 318 were excluded due to insufficient clinical information, 265 due to being Crosby capsule biopsies and 60 due to specific non GIFA indications (congenital diarrhoea, autoimmune enteropathy, graft-versus-host disease, tufting enteropathy and disaccharidase deficiency), leaving 676 patients who met the inclusion criteria. Some patients had multiple endoscopies and repeat biopsies, 122 in total, which were also excluded and only the initial presentation biopsy included. 554 endoscopic biopsy series were therefore included. Fifty-one per cent (285/554) were male, median age 7 months (IQR = 0.2-12 months). Overall, 62 % (344/554) had abnormal mucosal biopsy findings. The median age of those with abnormal biopsy was significantly lower than those with normal findings (median 6.6 months versus median 7.5 months, P < 0.001).

The most common presenting symptoms as indications for endoscopy were reflux/vomiting (40 %), faltering growth (37 %), diarrhoea (35 %) and rectal (PR) bleeding (12 %). 309 (56 %) patients presented with one symptom, 190 (34 %) with two, 49 (9 %) with three and six patients (1 %) with four symptoms. Positive predictive values (PPV) for symptoms based on the patients who presented with one symptom (*n* = 309) are shown in Table 1.

**Table 1:** Positive Predictive Values (PPV) of abnormal mucosal biopsies in infants being assessed for GIFA based on a single presenting gastrointestinal symptom based on 309/554 patients who presented with one symptom only

	Number	Percent	Isolated increased mucosal eosinophil density	PPV	Any abnormal biopsy	PPV
			*n*		*n*	
Reflux/vomiting	117	37.9 %	19	16.2 %	53	45.3 %
Diarrhoea	72	23.3 %	30	41.7 %	51	70.8 %
Faltering growth	61	19.7 %	19	31.1 %	31	50.8 %
PR Bleeding	31	10.0 %	16	51.6 %	23	74.2 %
Haematemesis	7	2.3 %	1	14.3 %	4	57.1 %
Constipation	6	1.9 %	3	50.0 %	3	50.0 %
Feeding difficulties	4	1.3 %	0	0.0 %	0	0.0 %
Irritability	4	1.3 %	1	25.0 %	2	50.0 %
Anaemia	2	0.6 %	0	0.0 %	0	0.0 %
Hypoalbuminaemia	2	0.6 %	0	0.0 %	2	100.0 %
Abdominal distension	2	0.6 %	1	50.0 %	1	50.0 %
Recurrent Abdominal pain	1	0.3 %	0	0.0 %	1	100.0 %

Diarrhoea was associated with a significantly greater frequency of abnormal histological findings than faltering growth (70.8 % vs. 50.8 %, *p* = 0.018) or reflux (70.8 % vs. 45.3 %, *p* = 0.001). PR bleeding was associated with a significantly greater rate of abnormal histological findings than faltering growth (74.2 % vs. 50.8 %, *p* = 0.031) or reflux (74.2 % vs. 45.3 %, *p* = 0.004). There were no significant differences between the frequency of abnormal biopsies between those presenting with diarrhoea and PR Bleeding, (*p* = 0.728) or those presenting with reflux and faltering growth, (*p* = 0.484).

Multiple logistic regression models were used to assess the frequency of any abnormal biopsy findings with combinations of symptoms (Hosmer-Lemeshow *p* = 0.373), as well as the probability of increased mucosal eosinophil density (Hosmer-Lemeshow *p* = 0.413), adjusted for differences in age and gender. Diarrhoea (*p* < 0.001), PR bleeding (*p* < 0.01), irritability (*p* < 0.05) and urticaria (*p* < 0.05) were significantly associated with both abnormal biopsy and excess eosinophils (Table 2). The site, Oesophagus, stomach, duodenum or colon, of the abnormal findings are shown in Table 3. Faltering growth, constipation and abdominal distension were also predictors of finding eosinophils in a biopsy (*p* < 0.05, *p* < 0.01, *p* < 0.05 respectively; Table 2). Age was an important confounding factor as we found a higher probability of an abnormal biopsy in younger children. Among others, reflux/vomiting was a poor predictor; therefore it was excluded from the models. Similarly, gender was not a significant confounding factor.

**Table 2:** Multiple logistic regression models for association with abnormal mucosal biopsies in infants being assessed for GIFA

	Regression model for excess eosinophils in biopsy		Regression model for any Abnormal Biopsy	
Variables in the model	B	*p*-value	B	*p*-value
Constant	−0.96	0.001	0.49	0.05
Age (months)	−0.07	0.033	−0.06	0.041
Urticaria	1.50	0.007	1.59	0.038
Irritability	1.69	<0.001	1.22	0.018
PR Bleeding	1.12	< 0.001	1.00	0.002
Diarrhoea	0.79	< 0.001	0.71	< 0.001
Constipation	1.42	0.002	n/a	n/a
Abdominal Distension	1.24	0.045	n/a	n/a
Faltering growth	0.49	0.016	n/a	n/a

**Table 3:** Symptoms related to the site of abnormal findings in cases with only a single site affected

	Oesophagus (*n* = 61)	Percent	Stomach (*n* = 16)	Percent	Duodenum (*n* = 50)	Percent	Colon (*n* = 44)	Percent
Reflux/Vomiting	44	72 %	5	31 %	19	38 %	15	34 %
FTT	18	30 %	5	31 %	26	52 %	18	41 %
Diarrhoea	5	8 %	6	38 %	19	38 %	18	41 %
PR Bleeding	3	5 %	0	0 %	5	10 %	10	23 %
Constipation	0	0 %	2	13 %	3	6 %	8	18 %
Anaemia	0	0 %	0	0 %	0	0 %	2	5 %
Feed Diff	2	3 %	2	13 %	3	6 %	2	5 %
Mouth Ulcers	0	0 %	0	0 %	0	0 %	0	0 %
Rash	1	2 %	0	0 %	3	6 %	2	5 %
Irritability	2	3 %	1	6 %	4	8 %	5	11 %
Haematemesis	6	10 %	3	19 %	1	2 %	0	0 %
Hypoalbuminaemia	0	0 %	1	6 %	2	4 %	1	2 %
Abd. Distension	1	2 %	0	0 %	1	2 %	5	11 %
RAP	1	2 %	0	0 %	0	0 %	3	7 %

Specific symptom combinations were more likely to have biopsies with excess eosinophils present. For example, children who presented with a combination of diarrhoea, PR bleeding and urticaria had an 88 % frequency of excess eosinophils.

## Discussion

This is the first large study to examine whether specific symptoms at presentation in very young children are associated with abnormal endoscopic biopsy findings in children being assessed for GIFA. The findings demonstrate that younger infants are more likely to have abnormal mucosal histological findings, and those presenting with specific combinations of symptoms are associated with high frequency of abnormal mucosal biopsy findings, including increased mucosal eosinophil density. In the current patient population, first presentation of GI symptoms occurred at around five months of age. However, it is likely that this represents a highly selected group referred to a specialist centre who are likely to have been experiencing more severe symptoms and hence were evaluated earlier in life than the general population and were all deemed to have symptoms of sufficient severity to warrant endoscopic examination. The risks of undergoing a general anaesthetic procedure and associated potential complications involved in performing endoscopy in very young infants as well as the impact on families are important considerations when deciding on whether to perform an endoscopy [13, 14, 19].

The most common symptoms were diarrhoea, reflux/vomiting, PR bleeding and faltering growth. Of these, if isolated, infants presenting with diarrhoea or PR bleeding, had an approximate 70 % probability of a histologically abnormal biopsy. However, infants who presented with combinations of diarrhoea, PR bleeding, irritability and urticaria were both more likely to have abnormal biopsy findings and also more likely to demonstrate increased mucosal eosinophil density. For example, almost 90 % of those presenting with diarrhoea and irritability had an abnormal biopsy. There were few patients who presented with irritability in isolation (1.3 %). However, it is possible that parents and clinicians underreport this symptom especially in the presence of other more common and recognisable symptoms. [20, 21] The frequency of an abnormal biopsy was lower for infants presenting with reflux/vomiting (45 %) and faltering growth (51 %).

Food allergic diagnoses are classified according to the site and severity of inflammation, which influences the presenting symptoms. With gastrointestinal mucosal disease that is identified by endoscopic biopsy there is a close spatial relationship of inflammatory mediators known to be released by mucosal inflammatory cells and enteric nerves [2]. The exact mechanisms of the manifestations of gastrointestinal symptoms are slowly being unravelled with the concept of paracrine immune interaction on the enteric nervous system, being known as a neuro-immune interaction [22, 23] leading to the disturbed motility and symptoms seen in GIFA such as reflux, diarrhoea or constipation. Much workstill needs to be done to fully explain how these symptoms develop and respond to dietary or anti-inflammatory measures.

Limitations of the study are related to the retrospective nature of data collected over a long time period with possible associated variation in the clinical suspicion of GIFA and management of such infants by endoscopic examination and biopsy. In GIFA, despite adherence to the diagnosis only being made by clinicians in out unit following elimination diet of major dietary antigens (Diary, egg, soya and wheat usually) with clinical improvement and subsequent reappearance of symptoms on rechallenge, the diagnosis remains subjective. The symptoms can be delayed and is unblended and subject to parental reporting. Furthermore, even with such a large dataset, for the purposes of this study we have classified mucosal biopsy findings broadly into normal versus abnormal, (with the only subcategory being those with apparently isolated increased mucosal eosinophil density at any site since this has been suggested as the most characteristic feature of GIFA) [12, 24]. More detailed sub-analysis of the relationship between other specific histological findings and their combinations with symptoms is not possible in this dataset and much larger numbers of cases, all of whom undergo multiple biopsies from small and large intestinal sites, would be required, but is unlikely to be available.

The clinical decision regarding whether an infant requires endoscopic examination and biopsy for diagnosis of food allergy can be difficult, since the procedure in this age group requires general anaesthesia with associated risks. The current data demonstrates that specific symptom patterns at presentation are associated with varying yield of abnormal mucosal histological findings, in particular, infants who experience diarrhoea, PR bleeding, irritability and urticaria having a high frequency of abnormal biopsies. This information may aid the decision making process for young children presenting with probable food allergy.

## Conclusions

Gastrointestinal food allergy (GIFA), may present with a wide variety of symptoms in the first year of life and specific symptom patterns at presentation are associated with varying yield of abnormal mucosal histological findings at endoscopic biopsy. Infants who experience diarrhoea, PR bleeding, irritability and urticaria have a high frequency of abnormal gastrointestinal mucosal biopsies, including prominent mucosal eosinophils.
